# Whole genome resequencing and phenotyping of MAGIC population for high resolution mapping of drought tolerance in chickpea

**DOI:** 10.1002/tpg2.20333

**Published:** 2023-04-30

**Authors:** Mahendar Thudi, Srinivasan Samineni, Wenhao Li, Martin P. Boer, Manish Roorkiwal, Zuoquan Yang, Funmi Ladejobi, Chaozhi Zheng, Annapurna Chitikineni, Sourav Nayak, Zhang He, Vinod Valluri, Prasad Bajaj, Aamir W. Khan, Pooran M. Gaur, Fred van Eeuwijk, Richard Mott, Liu Xin, Rajeev K. Varshney

**Affiliations:** ^1^ Center of Excellence in Genomics and Systems Biology (CEGSB) International Crops Research Institute for the Semi‐Arid Tropics (ICRISAT) Patancheru India; ^2^ Department of Agricultural Biotechnology and Molecular Biology Dr. Rajendra Prasad Central Agricultural University (RPCAU) Pusa India; ^3^ Crop Improvement Program—Asia, ICRISAT Patancheru India; ^4^ International Center for Biosaline Agriculture Dubai United Arab Emirates; ^5^ Wageningen University and Research Wageningen The Netherlands; ^6^ Khalifa Center for Genetic Engineering and Biotechnology (KCGEB) United Arab Emirates University Al Ain United Arab Emirates; ^7^ BGI‐Shenzhen Shenzhen China; ^8^ Department of Genetics, Evolution and Environment Genetics Institute, University College London London UK; ^9^ The UWA Institute of Agriculture University of Western Australia Perth Western Australia Australia; ^10^ Centre for Crop & Food Innovation, WA State Agricultural Biotechnology Centre Food Futures Institute, Murdoch University Murdoch Western Australia Australia

## Abstract

Terminal drought is one of the major constraints to crop production in chickpea (*Cicer arietinum* L.). In order to map drought tolerance related traits at high resolution, we sequenced multi‐parent advanced generation intercross (MAGIC) population using whole genome resequencing approach and phenotyped it under drought stress environments for two consecutive years (2013–14 and 2014–15). A total of 52.02 billion clean reads containing 4.67 TB clean data were generated on the 1136 MAGIC lines and eight parental lines. Alignment of clean data on to the reference genome enabled identification of a total, 932,172 of SNPs, 35,973 insertions, and 35,726 deletions among the parental lines. A high‐density genetic map was constructed using 57,180 SNPs spanning a map distance of 1606.69 cM. Using compressed mixed linear model, genome‐wide association study (GWAS) enabled us to identify 737 markers significantly associated with days to 50% flowering, days to maturity, plant height, 100 seed weight, biomass, and harvest index. In addition to the GWAS approach, an identity‐by‐descent (IBD)‐based mixed model approach was used to map quantitative trait loci (QTLs). The IBD‐based mixed model approach detected major QTLs that were comparable to those from the GWAS analysis as well as some exclusive QTLs with smaller effects. The candidate genes like *FRIGIDA* and *CaTIFY4b* can be used for enhancing drought tolerance in chickpea. The genomic resources, genetic map, marker‐trait associations, and QTLs identified in the study are valuable resources for the chickpea community for developing climate resilient chickpeas.

## Core Ideas

- Sequencing and analysis of MAGIC population in chickpea identified 932,172 SNPs, 35,726 deletions, and 35,973 insertions.
- A high‐density genetic map using 9195 SNPs covering a map distance of 1606.69 cM was developed.
- GWAS analysis identified 737 significant MTAs associated with key drought tolerance related traits.
- Major QTLs using an IBD‐based mixed model approach that were firstly computed using whole genome and pedigree information and then used as genetic predictors.

**Abbreviations**
CMLM: compressed mixed linear model
GWAS: genome wide association studies
IBD: identity‐by‐descent
MAGIC: multi‐parent advanced generation intercross

## 1 INTRODUCTION

Chickpea (*Cicer arietinum* L.) is the second most important grain legume cultivated largely on residual soil moisture especially in the semi‐arid regions of Sub‐Saharan Africa and South Asia. Globally, it is grown on 14.56 million ha with a total annual production of 14.76 million tons (FAOSTAT, 2018). It is an important source of protein, minerals, fiber, and vitamins in the diets of millions of people in Asia and Africa. Chickpea production is adversely affected by several abiotic and biotic stresses (Roorkiwal et al., 2020). During the last two decades, advances in genomics provided greater insights into understanding the genetics of complex traits. The most common way to dissect quantitative trait loci (QTLs) in several crop species is the use of populations derived from biparental crosses (Varshney et al., 2015). In the case of chickpea, several biotic and abiotic stresses, as well as agronomically important traits, have been mapped using biparental mapping populations (Barmukh et al., 2021; Jha et al., 2021; Mallikarjuna et al., 2017; Paul et al., 2018; Sab et al., 2020; Sabbavarapu et al., 2013; Soren et al., 2020; Varshney et al., 2019; Varshney, Thudi, et al., 2014).

Drought being a complex trait, both linkage mapping and linkage disequilibrium (LD)‐based mapping approaches were used in past (Thudi et al., 2014; Varshney et al., 2019; Varshney, Thudi, et al., 2014). Nevertheless, the availability of genome sequence (Varshney, Song, et al., 2013) and germplasm sequence information (Thudi, Chitikineni, et al., 2016; Thudi, Khan, et al., 2016; Varshney et al., 2021) enabled fine mapping of drought tolerance (Jaganathan et al., 2015; Kale et al., 2015). Besides understanding the genetics of the complex traits, during the last decade, molecular breeding approach like marker‐assisted backcrossing has been extensively used for introgression of QTLs into elite lines and molecular breeding lines with enhanced drought tolerance (Bharadwaj et al., 2021; Varshney, Gaur, et al., 2013), resistance to Fusarium wilt (Bharadwaj et al., 2022; Pratap et al., 2017; Mannur et al., 2019) and Ascochyta blight have been developed and released for commercial cultivation (Varshney, Mohan, et al., 2014). In addition, MutMap populations were also used to identify the causal SNPs and candidate genes in chickpea (Manchikatla et al., 2021). Owing to the use of two parental lines, QTLs identified by using the linkage mapping approach are of low resolution and are not necessarily validated in other genetic backgrounds. Although LD‐based mapping using natural populations overcomes this constraint, missing heritability, and the role of rare alleles remain unexplained. This situation is more serious in crops like chickpea which suffers with narrow genetic diversity. Hence, there is a great need to broaden the genetic base by incorporating maximum genetic variation available in the germplasm by involving multiple parents.

In recent years, specialized mapping populations that involve crossing of several parental lines, such as multi‐parent advanced generation intercross (MAGIC) (Cavanagh et al., 2008) and nested association mapping (Yu et al., 2008), are being used for genetic dissection of complex traits. Ever since the development of MAGIC populations in *Arabidopsis thaliana* (Kover et al., 2009), wheat (Huang et al., 2012), several research groups developed MAGIC populations in different crop species to understand the genetic architecture of several traits (Gangurde et al., 2022; Scott et al., 2020). Among multi‐parent populations, MAGIC populations serve as a great resource for understanding the genomic structure and provide a platform for a community‐based approach for gene discovery, characterization, and deployment of genes for understanding complex traits and improving the breeding populations (Huang et al., 2015; Scott et al., 2020).

In this study, we report fine mapping of drought tolerance using the desi chickpea MAGIC population derived from eight elite chickpea genotypes (ICC 4958, ICCV 10, JAKI 9218, JG 11, JG 130, JG 16, ICCV 97105, and ICCV 00108) (Samineni et al., 2021). The genomic resources, genetic map, and marker‐trait associations (MTAs) developed and identified in the study are valuable resources for chickpea community for developing climate resilient chickpeas.

## 2 MATERIALS AND METHODS

### 2.1 Plant material

Desi chickpea MAGIC population developed by Samineni et al. (2021) from eight founder parents (ICC 4958, ICCV 10, JAKI 9218, JG 11, JG 130, JG 16, ICCV 97105, and ICCV 00108) was used in this study for high resolution mapping of drought tolerance. In brief, 1136 F_7_ MAGIC lines were derived from eight founders through 28 two‐way, 14 four‐way, and 7 eight‐way crosses followed by six generations of selfing using single seed descent method (Samineni et al., 2021).

### 2.2 DNA isolation, library construction, sequencing, and variant calling

DNA was isolated from 15 day old seedlings using the high throughput mini‐DNA extraction method (Cuc et al., 2008). For whole genome resequencing (WGRS) of MAGIC population, we constructed libraries with 500 bp insert size for all samples as described in Varshney et al. (2017; Table S1). The high‐quality genomic DNA samples were randomly fragmented into fragments with a base pair peak of 300–700 bp. DNA fragments were then subjected to end repair (3′ and 5′) to generate blunt‐end, phosphorylated molecules. A single “A” base was added (A‐tailing) to the 3′ end of the end‐repaired, and adapters were ligated to both ends of the resulting fragments. The adapter‐ligated templates were purified by the Agencourt AMPure SPRI beads. The fragments with insert size about 500 bp were excised after separation on an agarose gel and then followed by PCR amplification. Finally, each library was loaded on Illumina HiSeq 2500 and sequenced to generate paired end reads.

The data of each line were cleaned and trimmed. SOAPnuke v1.5.6 was used to filter the raw reads of each line with parameters “filter ‐p 0.05 ‐n 0.1 ‐l l ‐q 0.2 ‐f AGATCGGAAGAGCACACGTCTGAACTCCAGTCAC ‐r AGATCGGAAGAGCGTCGTGTAGGGAAAGAGTGTA ‐S.” A series of checking and filtering measures on reads like (i) reads with more than 5% of bases or polyA structure; (ii) low quality reads (reads that have 20 bases with quality score less than or equal to 7 for each library data); (iii) adapter contamination; (iv) small insert size reads were filtered. Further, low quality bases at the end of the reads were trimmed off. The cleaned data were aligned on to the CDC Frontier genome (Varshney, Song, et al., 2013) using BWA (Li & Durbin, 2009) and sorted using Samtools v 0.1.18 (Li et al., 2009). The sorted BAM file is then passed through duplicate removal and local realignment using the command “MarkDuplicates” of Picard and “IndelRealigner/RealignerTargetCreator” of GATK v3.3‐0 with default parameters, separately.

**Core Ideas**

- Sequencing and analysis of MAGIC population in chickpea identified 932,172 SNPs, 35,726 deletions, and 35,973 insertions.
- A high‐density genetic map using 9195 SNPs covering a map distance of 1606.69 cM was developed.
- GWAS analysis identified 737 significant MTAs associated with key drought tolerance related traits.
- Major QTLs using an IBD‐based mixed model approach that were firstly computed using whole genome and pedigree information and then used as genetic predictors.

Variant calling was first executed using Samtools (Li et al., 2009)‐mpileup and the SNPs reported from Samtools were further used as known SNPs for variants calling through the Genome Analysis ToolKit (GATK v2.) pipeline. Variant calling pipeline refers to the “GATK Best joint genotyping Practices” workflow, followed by filtration using VariantFiltration with parameters “QD < 2.0 || MQ < 40.0 || ReadPosRankSum < −8.0 || FS > 60.0 || HaplotypeScore > 13.0 || MQRankSum < −12.5.” A position was reported as a variant with phred quality score >100 and if the reads aligned in each of the lines against the reference >5. The synonymous and non‐synonymous of SNPs were annotated using SnpEff v4.3t with default parameters.

### 2.3 Population structure and LD decay

SNPs with missing rate <0.8 and minor allele frequency >0.05 were considered using VCFtools v0.1.13 with parameters “–max‐missing 0.8 –maf 0.05.” ADMIXTURE (v1.3.0) with parameters “–cv ‐s time” was utilized for inferring population structure. LD decay statistics were calculated using software PopLDdecay (v3.41) with default parameters and were plotted using the corresponding script *Plot_MutiPop.pl* within the software.

### 2.4 Genetic map construction

SNPs with missing fraction >0.5 and segregation distortion at a stringent significant level ($1.5\times 10^{-23}$) were removed. MagicMap of RABBIT (v3.0) (Zheng et al., 2019) was used for genetic map construction. MagicMap first groups duplicate or co‐segregating markers into bins; the markers with the lowest number of missing data were used to represent each marker bin. The genetic map for these so‐called representative markers was obtained by pairwise linkage analysis, initial map construction via spectral grouping and ordering, and map refinement via simulated annealing. The final map was enriched by assigning all markers belonging to a particular bin to the position of the representative marker for that bin, whereas the ordering of the markers within a bin is non‐identifiable.

### 2.5 GWAS analysis

We performed the genome‐wide association study (GWAS) analysis using sequencing data generated on 8 founder parents and 1092 MAGIC lines (excluded the lines with insufficient data) and phenotyping data generated using augmented design earlier by Samineni et al. (2021) on the lines for two seasons (2013–14 and 2014–15). For GWAS analysis, a total of 262,515 SNPs found to be common in parents and population. To reduce the number of non‐informative SNPs from the analysis SNPs which are monomorphic among parents were excluded. Further, all heterozygous calls were considered missing. Using the PLINK analysis toolset three filters were applied (i) SNPs with >20% missing among MAGIC lines were excluded; (ii) SNPs with minor allele frequency ≤5% were also excluded; (iii) Individuals with >20% missing values were excluded. Finally, 1092 MAGIC lines comprising 126,275 SNPs were used for GWAS analysis. The compressed mixed linear model (CMLM) was implemented using GAPIT Version1 (Lipka et al., 2012) to establish MTAs. The reduced kinship matrix and three principal components were generated by PCA analysis through GAPIT. Both Kinship and PCA were included in the CMLM model to control the population structure and individual relatedness. The cutoff *p*‐value for significantly associated SNPs is set as 9.18 × 10^−08^.

### 2.6 QTL mapping using the IBD‐based mixed model approach

In addition to GWAS analysis that is based on IBS, we also performed QTL analysis based on identity‐by‐descent (IBD). We expect that major QTLs will be picked up by both approaches, but that both types of analysis may identify some interesting QTLs uniquely. To avoid co‐located markers and speed up the IBD‐based QTL analysis, we selected IBDs of every 10th position on the genetic map (Zheng et al., 2019, 2015) as genetic predictors, which led to 5718 positions for a genome wide scan. We used the IBD‐based multi‐QTL model (IBD.MQM) (Li et al., 2021) to identify QTLs whose effects were modeled as random effects. A likelihood ratio test (LRT) was used to evaluate variance components representing potential QTLs. LRTs were transformed to *p*‐values using a $0.5\chi_{0}^{2}+0.5\chi_{1}^{2}$ mixture distribution (Self & Liang, 1987). The *p*‐value corresponding to the LRT statistic was expressed as a −log10(*p*). A simple Bonferroni threshold was used to deal with the multiple‐testing issue in the IBD‐based mixed model approach (Li et al., 2021). Therefore, we used the threshold 5.7 from the Bonferroni‐adjusted threshold placed at a genome‐wide significance level of 0.01 for 5718 positions. To select cofactors, an exclusion window of 30 cM around cofactors was set within which no test for further QTLs was performed. We performed multiple genome scans with cofactors until no further significant QTLs were detected. Parental effects at QTL candidates were estimated in a final model containing all earlier identified QTLs.

## 3 RESULTS

### 3.1 Whole genome resequencing (WGRS) of founder lines and their population

A total of 52.02 billion clean reads (90 bp) containing 4.67 TB clean data were generated on the MAGIC population comprising 1136 lines and 8 founder lines (ICC 4958, ICCV 10, JAKI 9218, JG 11, JG 130, JG 16, ICCV 97105, and ICCV 00108) were generated using the WGRS approach on Illumina HiSeq 2500 (Table S1). On an average, the sequencing depth on founder lines was 48.74×, and 96.52% of the reads generated were aligned with 98.81% genome coverage (Table S2). The lines built up MAGIC groups of 105 to 217 lines with an average size of 162 lines. The clean reads obtained on each MAGIC line varied between 2.54 and 8.38 Gb. Further, on an average the sequencing depth on MAGIC lines was 6.97×, and 96.56% of the reads generated were aligned with 92.63% genome coverage (Table S3).

### 3.2 Distribution of variations in the genome

In total, 932,172 SNPs were identified on aligning the MAGIC lines to the CDC Frontier reference genome (Table 1; Varshney, Song, et al., 2013). The average SNP density was maximum (4.38 SNPs/kb) in case of Ca4 and minimum (0.99 SNPs/kb) in case of Ca5. The number of SNPs varied from 96,600 to 249,018 with an average of 148,112.89 per individual MAGIC line (Table S4). The mean proportion of heterozygote allele calls among the founder lines ranged from 36% to 47%, unexpected high level of heterozygote allele calls of 47% was observed (Table S5). In case of common bean (*Phaseolus vulgaris*), 19.6% of heterozygote allele calls were reported in MAGIC population, which was also unexpected high level of heterozygosity (Diaz et al., 2020). Pairwise SNPs between founder lines ranged from 660,074 (between JG 130 and JAKI 9218) to 971,540 (between JG 11 and ICCV 97105) (Table S6).

**TABLE 1:** Summary of genome wide variation in multi‐parent advanced generation intercross (MAGIC) population.

Pseudomolecule	Deletions	Insertions	SNPs	Average SNP density per kb	Total variant count	Synonymous SNPs in genes	Non‐synonymous SNPs in genes	Total SNPs in genes	Average variant density per kb
Ca1	4857	4724	112,443	2.53	122,024	2800	2769	20,116	2.75
Ca2	2542	2659	56,752	1.71	61,953	1423	1399	9611	1.87
Ca3	1978	2084	46,239	1.23	50,301	1086	1331	7936	1.34
Ca4	9070	8966	203,540	4.38	221,576	3784	3552	28,675	4.77
Ca5	1736	1741	43,337	0.99	46,814	764	1132	5885	1.07
Ca6	3725	3884	79,836	1.46	87,445	1818	1911	12,184	1.60
Ca7	3180	3347	67,025	1.46	73,552	1264	1496	9707	1.60
Ca8	1289	1261	25,617	1.77	28,167	674	663	5401	1.94
Ca0	7349	7307	297,383	1.82	312,039	1579	2996	12,371	1.91
Total	35,726	35,973	932,172		1,003,871	15,192	17,249	111,886	

In total 111,886 SNPs in genes were identified, of which 17,249 were non‐synonymous SNPs and 15,192 were synonymous (Table 1). Further, the number of SNPs identified in genes varied from 5401 to 28,675. The number of synonymous SNPs in genes varied between 674 and 3784, whereas non‐synonymous SNPs varied between 663 and 3552. On an average 86.57% SNPs were identified in intergenic regions. Among the SNPs in intragenic region, the SNPs in intron were high (varied between 14,150 and 31,943 with an average of 17932.42) compared to exon (Table S4). In total, we identified 35,726 deletions and 35,973 insertions among the MAGIC population (Table 1). Like SNPs, the distribution of insertions and deletions varied greatly on the eight pseudomolecules and maximum number of insertions (8966) and deletions (9070) were present on Ca4 and minimum number on Ca8 (1261 insertions and 1289 deletions).

#### 3.2.1 Founder specific unique SNPs

In total we identified 57,325 founder specific unique SNPs in all eight founder liness used for developing MAGIC population. Among the founder lines, we identified large number of unique SNPs in case of ICCV 97105 (20,592 SNPs) and least number of SNPs in the founder lines JAKI 9218 (147 SNPs; Table S7). Although Ca4 harbored large number of founder specific unique SNPs (16,436), none of the founder lines had largest number of SNPs on Ca4 except for ICCV 00108.

#### 3.2.2 Founder specific unique SNPs in MAGIC population

Of 57,325 founder specific unique SNPs only 32.38% (18,564 SNPs) were present in the MAGIC population. We observed large proportion of SNPs from founder lines ICCV 97105 (11,044) followed by JG 16 (9528), ICC 4958 (5716), ICCV 10 (2126), ICCV 00108 (1346), JG 11 (1121), JAKI 9218 (48), and JG 130 (25) (Table S7). Although Ca6 harbored large number of founder line specific unique SNPs (5686), except for ICC 4958 and JG 11, none of the founder lines had largest number of SNPs on Ca6. A haplotype analysis was performed to quantify the individual contribution from each founder line in the population. All eight parental genomes contributed to all pseudomolecules and each founder genome is expected to be represented by 12.5% in the population. The percentage of founder specific unique alleles transferred or found in the population varied from 15.82% (JG 130) to 89.4% (ICCV 10) with an average of 51.05% (Table S8). These values are higher than the expected contribution of 1/8th (12.5%).

### 3.3 Population structure and LD decay

We determined the population structure among the 1136 RILs of MAGIC population derived from seven eight‐way crosses using Admixture and identified 13 subpopulations (i.e., *K* = 13) (Figure S1A). Large proportion of genome reshuffling in the MAGIC population is evident from the allelic admixture among the seven groups derived from eight‐way crosses (Figure S1B). LD decay varied from 0.5 to 1.5 Mb (Figure S2). The mean LD for the genome decreased to *r* ^2^ < 0.2 within 1.2 Mb. In case of barley (*Hordeum vulgare*), genome‐wide LD decay varied between 14 and 38 Mbp (Novakazi et al., 2020). However, in the case of legumes like common bean, rapid LD (51 to 154 kb) was reported (Diaz et al., 2020).

### 3.4 High correspondence between physical and genetic maps

Excluding markers with a missing fraction >0.5 and segregation distortion resulted in a total of 57,180 SNPs, which were assigned to 13,172 marker bins. The resulting linkage map spanned 1606.69 cM across eight linkage groups (Figure S3; Table S9). The number of representative markers per linkage group ranged from 1561 (on CaLG03) to 14841 (on CaLG01). The lengths of the linkage groups ranged from 111.8 cM (Ca3) to 295.0 cM (Ca1). As expected, nonrepresentative co‐segregating markers, mostly in the regions with low recombination rates, were not well ordered. On aligning the physical and genetic maps we observed some rearrangements (inverse) of chromosome segments. The overall heatmap of the recombination fraction matrix showed a clear diagonal structure. High correspondence between physical and genetic map was observed for all linkage groups except CaLG01 and CaLG08 (Figure S3).

### 3.5 Genome‐wide association analysis

A large variation among MAGIC lines was reported for flowering time (34–68 days), maturity (101–120 days), plant height (31.5–65 cm), number of pods per plant (14–150), number of seeds per plant (14–186), seed yield (255–4555 kg/ha), harvest index (HI) (0.10–0.88), and seed size (11.2–39.3 g) under rainfed and irrigated conditions. After a series of filtration steps (Figure S4), a total of 126,275 SNPs and the phenotyping data generated during 2013–14 and 2014–15 were used to identify significant MTAs. A total of 737 significant MTAs (8.31 × 10^−33^ to 9.18 × 10^−08^) with 0.86%–10.35% phenotypic variation were identified during 2013–14, 2014–15, and pooled data (Table 2; Table S10). Notably 72.99% (538) of MTAs were identified on Ca8 and remaining on Ca4. MTAs for majority of the traits like plant height (PHT), days to 50% flowering (DF), days to maturity (DM), and biomass (BM) are identified on Ca8, except for 100 seed weight (100SDW) and HI for which MTAs were identified on both Ca4 and Ca8.

**TABLE 2:** Summary of genome wide marker‐trait association identified based on the SNPs and phenotyping data generated on multi‐parent advanced generation intercross (MAGIC) population during 2013–14 and 2014–15.

Trait	Year	Number of MTAs	*p* value	Phenotypic variation (%)
*Yield related traits*				
100 seed weight (g)	2013–14	69	8.31 × 10^−33^ to 9.18 × 10^−08^	0.9–4.74
	2014–15	65	1.28 × 10^−20^ to 9.31 × 10^−08^	1.32–4.13
	Pooled	70	1.02 × 10^−31^ to 8.35 × 10^−08^	0.86–4.37
Harvest index (%)	2013–14	14	1.73 × 10^−11^ to 5.48 × 10^−08^	2.64–4.08
	2014–15	56	1.54 × 10^−13^ to 8.73 × 10^−08^	2.43–4.68
	Pooled	50	2.06 × 10^−16^ to 9.8 × 10^−05^	2.30–5.56
Biomass (kg/ha)	2013–14	–	–	–
	2014–15	2	2.06 × 10^−08^ to 5.28 × 10^−08^	2.61–2.77
	Pooled	–	–	–
*Phenological traits*				
Days to 50% flowering	2013–14	67	2.26 × 10^−11^ to 9.91×10^−08^	2.51–3.98
	2014–15	64	1.8 × 10^−15^ to 9.48 × 10^−08^	2.53–5.71
	Pooled	63	5.89 × 10^−10^ to 8.36 × 10^−08^	2.75–3.45
Days to maturity	2013–14	14	1.20 × 10^−31^ to 9.2 × 10^−08^	2.05–10.35
	2014–15	91	2.74 × 10^−18^ to 9.47 × 10^−08^	2.42–6.66
	Pooled	29	2.62 × 10^−31^ to 6.6 × 10^−08^	2.01–9.79
*Morphological traits*				
Plant height (cm)	2013–14	33	6.59 × 10^−11^ to 6.33 × 10^−08^	2.66–3.90
	2014–15			
	Pooled	52	4.98 × 10^−13^ to 9.98 × 10^−08^	2.43–4.53

Abbreviation: MTAs, marker‐trait associations.

### 3.6 GWAS signal for yield related traits

#### 3.6.1 100 seed weight (100SDW)

**FIGURE 1:**
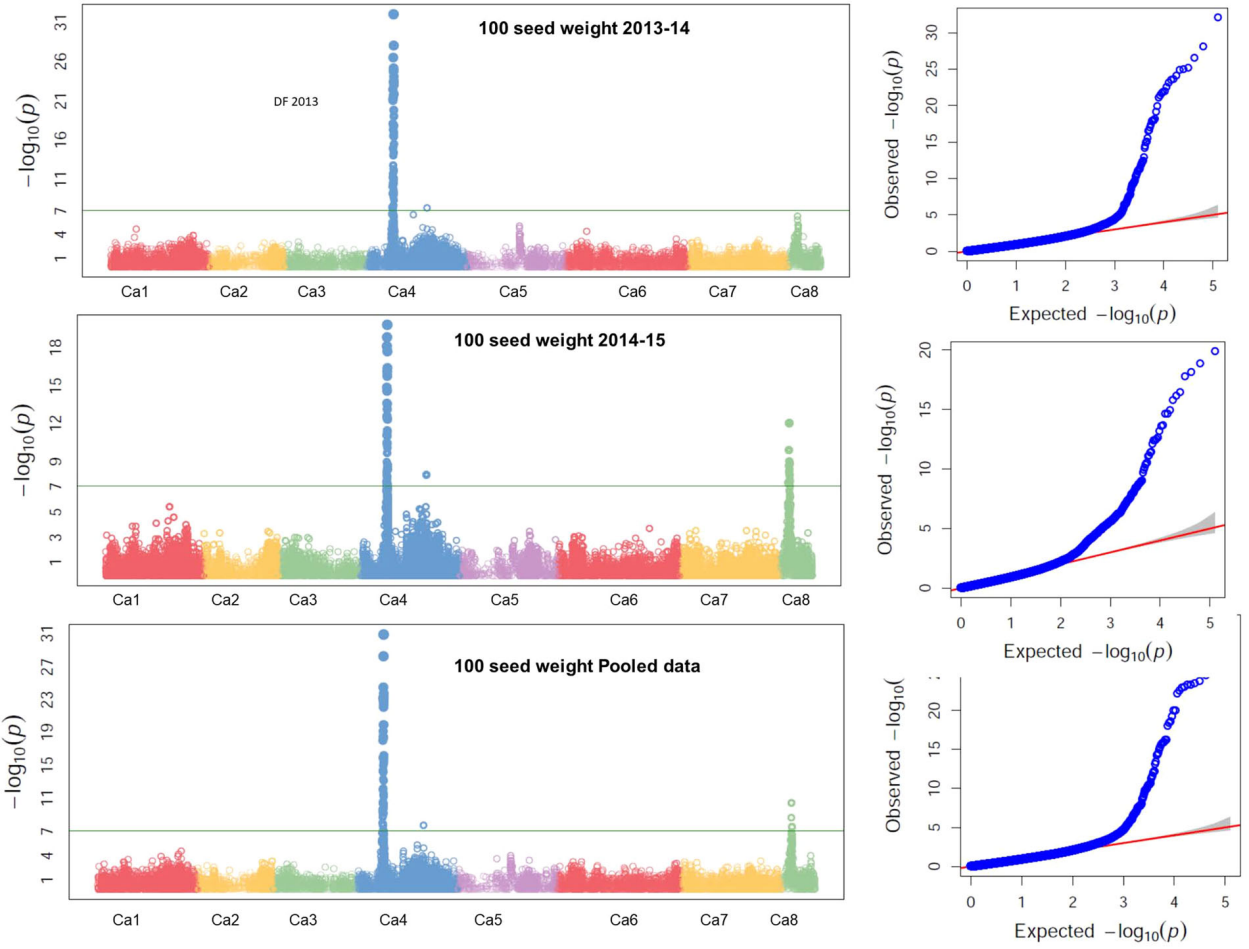
Genome wide association signals for 100 seed weight. Majority of associations for 100 seed weight are identified on Ca4 and some associations on Ca8. Among 204 marker‐trait associations (MTAs) identified for 100 seed weight 35 associations were stable (identified based on data generated during 2013–14, 2014–15, and pooled data from both seasons).

We identified a total of 204 significant MTAs for 100SDW. These MTAs explained 0.86%–4.74% phenotypic variation (Table 2). Of these, 106 MTAs were identified in more than one season and pooled data (Figure 1; Table S10). Majority of MTAs (89.21%) were identified on Ca4 remaining were present on Ca8. Thirty‐six MTAs were consistently detected in two seasons as well as in the pooled data. Among these, 73.56% MTAs were present in intragenic regions and the remaining were present in genic regions. Based on the physical position of MTAs, 54 MTAs were present in 22 unique genes. These genes encoded functions like LRR receptor‐like kinase family protein (Ca_04539), ATPase family AAA domain‐containing protein 3C (Ca_02032), DNA‐directed RNA polymerase III subunit RPC5 (Ca_02065), transmembrane ascorbate ferrireductase (Ca_04475), DNA mismatch repair MSH3‐like protein (Ca_04480), calmodulin‐binding protein (Ca_04493), PTI1‐like tyrosine‐protein kinase At3g15890 (Ca_04525), protein TIFY 4B‐like isoform X4 (Ca_04558), LRR/extensin 2 (Ca_04564), frigida‐like protein (Ca_04589), and so on (Table S10).

#### 3.6.2 Harvest index (HI) and biomass (BM)

In the case of HI, we identified 120 significant MTAs that explained about 2.30–5.56 phenotypic variation on Ca4 and Ca8 (Table 2). Among these 106 MTAs were identified based on 2014–15 data as well as pooled data from both years (Table S10). Fourteen significant MTAs were identified based on data generated during 2013–14 crop season. Among significant MTAs, 75.83% were identified in intergenic regions and remaining were present in either exonic or intronic regions of 17 genes (Table S10). In the case of BM, we identified only two significant MTAs that explained 2.61% to 2.77% phenotypic variation during crop season 2014–15.

#### 3.6.3 Phenological traits

**FIGURE 2:**
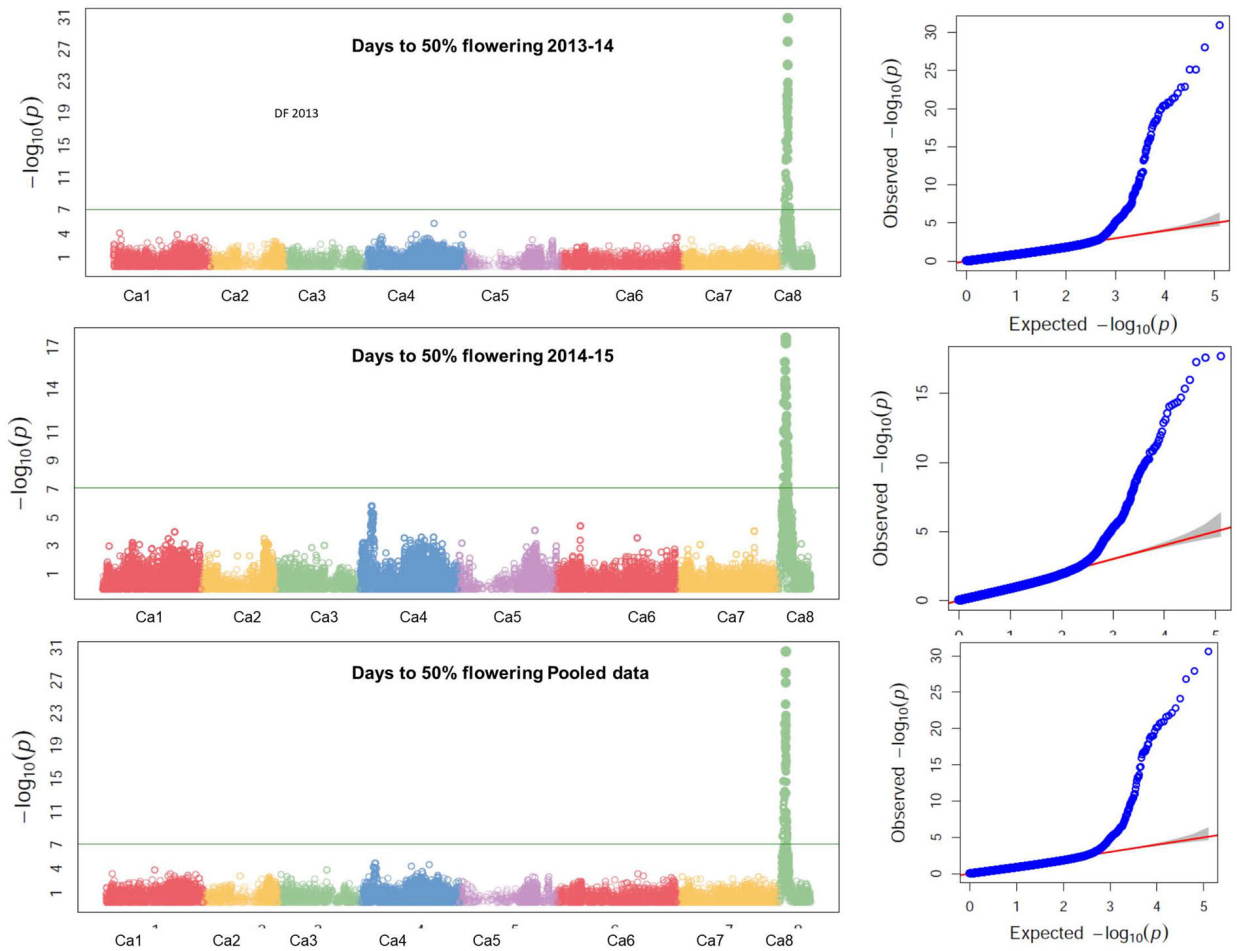
Genome wide association signals for days to 50% flowering. All marker‐trait associations (MTAs) were identified on Ca8. Among 194 MTAs identified for days to 50% flowering 36 associations were stable (identified based on data generated during 2013–14, 2014–15, and pooled data from both seasons).

**FIGURE 3:**
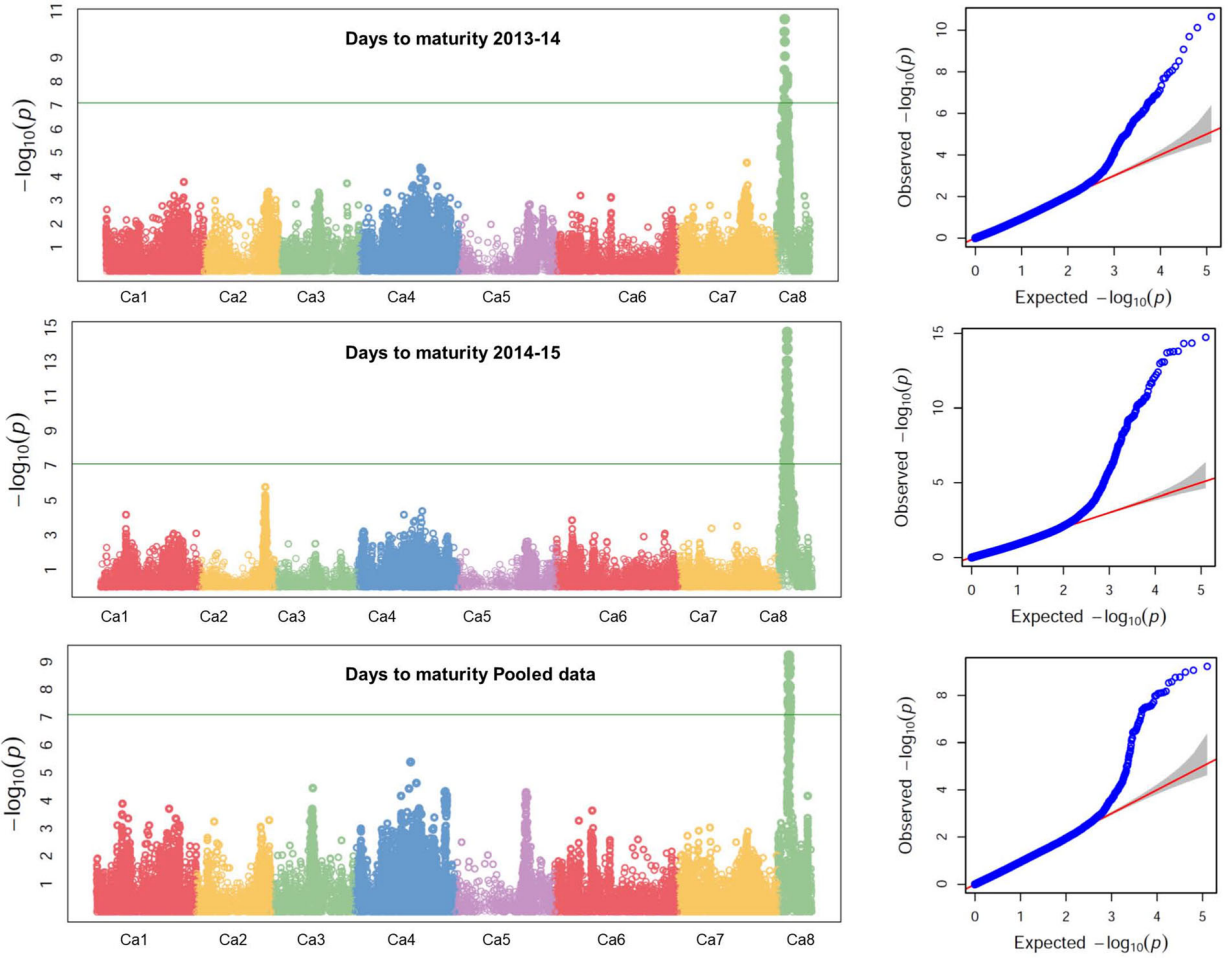
Genome wide association signals for days to maturity. All marker‐trait associations (MTAs) were identified on Ca8. Among 132 MTAs identified for days to maturity 21 associations were common based on data generated during 2013–14, 2014–15, and pooled data from both seasons.

Days to 50% flowering (DF) and days to maturity (DM) are phenological traits that have key role to play on chickpea yield. In the case of DF, we identified 194 MTAs that explained phenotypic variation ranging from 2.01% to 10.35%. Of these, 87 MTAs were unique and 65 were consistently detected in both seasons and pooled data (Figure 2; Table 2). Further, 31 MTAs were present in 13 genes (Table S10). In case of DM, a total of 132 MTAs were identified and explaining a phenotypic variation ranged from 2.51 to 3.98. Majority of MTAs (89) were identified during crop season 2014–15. Of these, 34 MTAs were consistently detected in both seasons and pooled data (Figure 3; Table S10). Among 132 MTAs, 105 were present in intergenic region and remaining were present in genic regions (15 exon and 12 intron).

#### 3.6.4 Morphological traits

For PHT a total of 85 MTAs were identified which explained phenotypic variation ranging from 2.43% to 4.53%. Of 85 MTAs, 52 were identified based on pooled data of 2013–14 and 2014–15 data. While remaining 33 MTAs were identified only during 2013–14. Nevertheless, 31 MTAs were commonly identified between pooled data and data generated during 2013–14 (Table 2). Based on physical position of SNPs, nine MTAs were identified in six genes (Ca_02021, Ca_02081, Ca_02158, Ca_02159, Ca_02202, and Ca_02216), whereas remaining MTAs were not in the genic region. Among these genes, the gene Ca_02216 encodes for formin‐like protein 6, Ca_02021 encodes for transmembrane protein 53‐like, Ca_02158 encodes for PI‐PLC X domain‐containing protein At5g67130, Ca_02159 encodes for receptor‐like kinase plant, Ca_02202 encodes for probable prolyl 4‐hydroxylase 9, and Ca_02081 encodes for E3 ubiquitin‐protein ligase RNF185‐like.

### 3.7 Identification of QTLs using IBD‐based mixed model approach

Using an IBD‐based mixed model approach as a complement to a GWAS approach, a total of 36 QTLs were identified for five traits. Among these 36 QTL, nine QTLs were major (>10% phenotypic variation) and 27 QTLs were minor (<10% phenotypic variation). Although maximum number of QTLs were identified for 100SDW trait (seven QTLs in 2013 and eight in 2014), none of the QTLs were stable, that is, appeared in both years. Among 15 QTLs, two QTLs (Ca4_12823696 and Ca4_13407573) on Ca4 explained maximum phenotypic variation, 29.58% and 37.9%, respectively. In case of DF, one stable QTL (Ca8_4123449) explaining 10%–11% phenotypic variation was identified on Ca8 based on both 2013–14 and 2014–15 phenotyping data (Table 3). However, the QTL identified (Ca2_32077608) on Ca2 explained 33.97% phenotypic variation for DF. A total of 5 QTLs (two QTLs in 2013 and three in 2014) were identified for DM, explaining phenotypic variation between 1.9% and 20.68%.

**TABLE 3:** Summary of quantitative trait loci (QTLs) identified using identity‐by‐descent (IBD) mixed model approach.

		QTLs			Parental effects								
Trait	Year	Name	Pseudo‐molecule	Position (cM)	ICCV 00108	ICCV 10	ICCV 97105	ICC 4958	JAKI 9218	JG 11	JG 130	JG 16	Phenotypic variation (%)
Days to 50% flowering	2013–14	Ca2_28110545	Ca2	64.775	−0.01	−0.47	−0.05	−0.63	2.46	−0.92	1.62	−2.00	6.98
		Ca3_22180448	Ca3	13.862	0.56	−0.21	−1.13	−0.06	0.96	0.72	−0.26	−0.59	2.19
		Ca6_8281762	Ca6	62.683	−0.43	−1.02	0.27	1.12	0.08	0.19	−0.41	0.20	1.87
		Ca8_4123449	Ca8	36.146	−0.92	1.37	0.60	−1.74	0.04	−2.15	−0.58	3.39	11.60
	2014–15	Ca1_43251792	Ca1	145.912	0.19	−0.03	0.00	−0.15	−0.18	−0.31	0.18	0.30	0.92
		Ca2_32077608	Ca2	71.286	−0.24	−0.68	−0.12	−0.22	3.75	−0.29	−1.37	−0.82	33.97
		Ca8_4123449	Ca8	36.146	−0.63	0.51	0.73	−0.12	−0.28	−0.84	−0.79	1.42	10.09
Days to maturity	2013–14	Ca2_32146004	Ca2	71.200	−0.97	−0.44	−0.08	−0.89	4.66	−0.67	−0.13	−1.48	18.66
		Ca8_4123449	Ca8	36.146	−1.01	1.69	0.81	−0.91	0.25	−1.53	−1.96	2.65	13.96
	2014–15	Ca2_12005466	Ca2	54.879	−0.73	−1.35	−1.25	−0.56	4.50	−1.12	1.57	−1.06	18.82
		Ca2_32389765	Ca2	70.996	−0.02	0.19	0.12	−0.33	0.82	0.45	−0.19	−1.04	1.90
		Ca8_4123449	Ca8	36.146	−1.34	2.02	1.46	−1.75	−1.01	−1.41	−1.77	3.79	20.68
Harvest index ( %)	2013–14	Ca8_4123449	Ca8	36.146	0.01	−0.02	−0.01	0.01	0.01	0.02	0.01	−0.03	3.73
	2014–15	Ca8_4123449	Ca8	36.146	0.01	−0.01	−0.01	−0.01	0.02	0.01	0.02	−0.04	5.61
100 seed weight (g)	2013–14	Ca1_13279659	Ca1	219.271	−0.19	−0.14	0.53	0.22	−0.15	0.32	−0.33	−0.26	0.98
		Ca4_13407573	Ca4	92.103	1.78	−1.63	2.26	2.20	−2.99	−1.93	2.32	−2.02	37.90
		Ca4_32458057	Ca4	139.735	0.20	0.03	−0.93	0.27	0.48	0.37	−0.04	−0.38	2.05
		Ca5_30240538	Ca5	16.675	−0.52	−0.12	0.04	0.25	0.01	0.39	−0.09	0.04	0.70
		Ca6_8354228	Ca6	61.069	0.21	0.16	−0.52	−0.06	0.20	0.37	−0.01	−0.36	1.03
		Ca7_2343256	Ca7	46.671	−0.10	−0.05	−0.14	0.59	−0.06	0.33	−0.25	−0.33	0.94
		Ca8_5063665	Ca8	50.079	−0.52	0.07	−0.26	−0.30	0.65	−0.16	0.40	0.12	1.38
	2014–15	Ca1_11574799	Ca1	242.772	−0.03	−0.21	0.09	−0.01	0.12	0.34	0.20	−0.51	0.81
		Ca3_37349467	Ca3	95.511	−0.44	−0.25	0.25	0.20	−0.05	0.22	−0.14	0.21	0.84
		Ca4_1466459	Ca4	15.196	−0.14	0.07	−0.44	−0.15	0.05	0.41	0.07	0.14	0.65
		Ca4_12823696	Ca4	91.424	1.78	−1.50	1.77	2.10	−2.41	−1.72	1.81	−1.82	29.58
		Ca4_32537409	Ca4	139.735	0.17	−0.06	−0.68	0.33	0.33	0.07	0.03	−0.18	1.19
		Ca5_6638621	Ca5	209.002	0.38	−0.14	0.47	−0.19	−0.48	−0.15	−0.43	0.53	1.74
		Ca6_15115420	Ca6	90.081	−0.12	−0.04	−0.38	0.06	0.36	0.48	0.18	−0.54	1.22
		Ca8_5063665	Ca8	50.079	−0.36	−0.20	−0.02	−0.01	0.53	−0.19	0.58	−0.33	1.30
Plant height (cm)	2013–14	Ca1_16017040	Ca1	198.500	−0.41	−0.17	0.12	−0.45	0.44	−0.29	1.38	−0.63	3.44
		Ca2_31949226	Ca2	85.866	−0.29	−0.18	0.45	−0.39	0.09	−0.09	−0.12	0.53	1.21
		Ca3_27203587	Ca3	25.719	0.87	−0.16	−0.76	−0.07	0.52	0.33	0.05	−0.78	3.09
		Ca4_13151927	Ca4	90.389	1.01	−1.00	1.09	1.04	−0.51	−1.08	0.52	−1.08	7.50
		Ca8_5294843	Ca8	51.099	−0.94	0.61	−0.19	−0.30	0.24	−0.44	0.40	0.61	2.64
	2014–15	Ca4_13151927	Ca4	90.389	0.63	−0.73	0.72	0.63	0.09	−0.84	0.52	−1.01	3.43
		Ca8_4311705	Ca8	37.288	−1.00	0.49	−0.36	−0.40	0.60	−0.66	0.52	0.81	3.29

**FIGURE 4:**
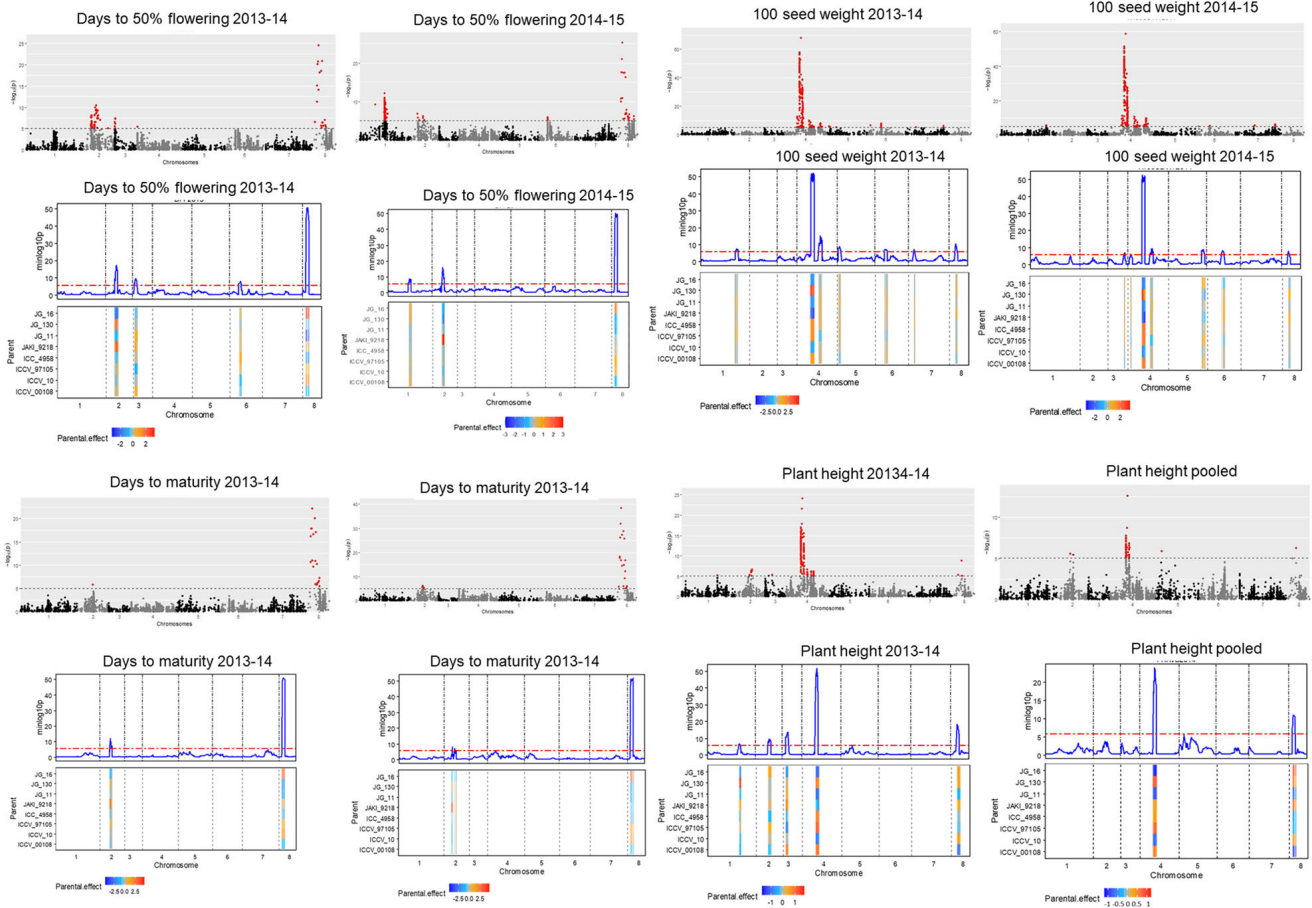
Quantitative trait locus (QTL) profiles and parental effects. The figure shows the QTL profiles (IBS vs. identity‐by‐descent [IBD] model) (upper panel) and parental effects by IBDs (lower panel) for each trait. For some traits (e.g., DF, DM, and 100 seed weight), the −log10(*p*) values of peak positions exceed 50. We scaled the *y*‐axis at the range from 0 to the maximum 50 to easily check mapping signals of other QTLs with relatively lower −log10(*p*) values. For some traits (e.g., BM and YLD2013), no evidence to show any significant QTL, and thus we did not show their mapping profiles.

Major QTLs for each trait identified by the IBD‐based mixed model (Figure 4) were confirmed by the GWAS results. For instance, the region of the strongest QTL on Ca4 for 100SDW over two seasons is comparable to those significant MTAs identified by GWAS, while the IBD‐based mixed model identified extra QTLs with relatively small effects on other chromosomes. A similar scenario is also observed in the mapping results for DF where the major QTL on Ca8 was found by both the IBD‐based mixed model and GWAS approaches, whereas some exclusive minor QTLs were found by the former approach. For DM, the IBD‐based mixed model identified one extra QTL on Ca2.

QTLs identified for different traits on eight chromosomes are shown in Figure 3. Ca4 and Ca8 locate multiple shared QTL regions for several traits. A QTL region (around marker Ca8_4123449, 36 cM) on Ca8 was identified for three traits (DF, DM, and HI), whereas parents at this shared QTL render similar effects on DF and DM that are distinct from HI (Table 3). On Ca4, the QTL region around 90–92 cM is found to be responsible for two traits (100SDW, PHT) with similar contributions from each parent.

## 4 DISCUSSION

For creating and harnessing the genetic diversity, several research groups during last decade developed MAGIC populations in different crops species like *Oryza sativa* (rice, Bandillo et al., 2013; Descalsota et al., 2018; Ogawa et al., 2018), *H. vulgare* (barley, Sannemann et al., 2015), *Gossypium hirsutum* (upland cotton, Islam et al., 2016) *Zea mays* (maize, Dell'Acqua et al., 2015), *Vicia faba* (faba bean, Sallam & Martsch, 2015), *Solanum lycopersicum* (tomato, Pascual et al., 2015), and *Sorghum bicolor* (sorghum, Ongom & Ejeta, 2018). MAGIC populations also enabled to understand the genetic architecture of several traits. Although the development of MAGIC populations is complex and resource intensive, due to their potential and interest for breeding, MAGIC populations are being developed involving 4–64 founder lines using different crossing schemes (Arrones et al., 2020). The size of the MAGIC populations varied depending on several factors like crossing scheme, the breeding behavior of the species. In the case of legumes, the size of *P. vulgaris* (common bean) MAGIC population was 996 RILs, whereas in the case of chickpea the size was 1136 RILs.

No clear population structure was observed in the MAGIC population we developed as the *K* value is 13. Based on a simulation model, the number of recombination events per RIL in a population is expected to increase with the number of founder lines and the number of crosses involved (Huang et al., 2012; Ladejobi et al., 2016). In general, large number of recombinations allowed during development of MAGIC populations will reduce the LD decay and make the population more useful for fine mapping of traits. However, in case of chickpea, LD decay reported based on 429 lines resequencing was faster than the one reported in this study. This may be due to the use of elite founder lines used for developing the population. Higher proportion of heterozygosity among founder lines found in this study is not uncommon. Similar higher heterozygosity was also reported in common bean (Diaz et al., 2020).

Earlier using QTL mapping approach, a genomic region (∼29 cM) on CaLG04 was reported to harbor QTLs for drought tolerance related traits. This genomic region that was referred as *QTL‐hotspot* was further fine mapped using GBS (Jaganathan et al., 2015) and skim sequencing approaches and the region was narrowed down to *QTL‐hotspot_a* and *QTL‐hotspot_b* spanning few kilobases (Kale et al., 2015). We fine mapped drought tolerance related traits in this study using MAGIC population. MTAs for 100SDW were mainly identified on Ca4 between 13024647 and 13761934 regions. Earlier 100SDW was reported in *QTL‐hotspot_a* that was between Ca4_13239546 and Ca4_13378761. Further, MTAs for different traits have been co localized on Ca8, for instance, DF, DM, HI, and PHT. Similarly, QTLs identified using IBD for 100SDW and PHT were also colocalized in the *QTL‐hotspot* region reported earlier. Similar colocalizations of QTLs were also reported in the case of *indica* rice; of 18 QTLs identified for the five‐grain size‐related traits, 12 QTLs were reported to be colocalized with the cloned genes (Ponce et al., 2020). Similarly, MAGIC population developed from eight Mesoamerican breeding lines of common bean enabled identification of a major *QTL‐hotspot* located on chromosome Pv01 for phenology traits and yield (Diaz et al., 2020).

GWAS conducted earlier using chickpea reference set reported robust (explaining >10% phenotypic variation), stable (more than one location), and consistent MTAs (more than one season) (Varshney et al., 2019). However, in this study we identified only consistent MTAs with small phenotypic variation. Nevertheless, the genes we identified in the present study based on the physical position of the MTAs play key role in drought tolerance mechanism. For instance, *FRIGIDA* gene identified in the present study was reported to activate *P5CS1* expression and promoting proline accumulation in the case of *A. thaliana* that enhances drought tolerance during water stress (Chen et al., 2018). Further, *FRIGIDA* gene upregulates the expression of the *FLOWERING LOCUS C* and accelerates transition to flowering after vernalization (Li et al., 2018). Another gene, TIFY 4B‐like isoform X4, present in plant vigor QTLs was reported to enhance the seed size and leaf size in chickpea (Nguyen et al., 2022). In addition, a recent study in chickpea also reported that a non‐synonymous substitution in the transcription factor *CaTIFY4b* regulates seed weight and organ size in chickpea (Barmukh et al., 2022). Further, characterization of *QTL‐hotspot* introgression lines revealed *CaTIFY4b*‐H2 haplotype as the superior haplotype for 100‐seed weight. Hence, markers can be designed to the SNP (Ca4_13331455) or the superior haplotype *CaTIFY4b*‐H2 can be used in early generation selection for seed weight in chickpea.

Similarly, the gene Ca_02267 that encodes for N‐acetylornithine deacetylase was reported to play major role in flowering and fruit development in Arabidopsis (Molesini et al., 2015). Another gene, Ca_02081 that encodes for E3 ubiquitin‐protein ligase RNF185‐like was extensively studied both at genomic and phenomic level in the case of Arabidopsis and was reported to regulate flowering time (Pavicic et al., 2017). The SNP Ca8_4764769, in the gene Ca_02158 associated with plant height encodes for PI‐PLC X domain‐containing protein At5g67130, however, was reported to be downregulated in soybean under salt stress (Rehman et al., 2022). The SNP Ca8_2686723, in the gene Ca_02310 associated with HI in the present study, encodes for glutamate dehydrogenase (GDH) 2, GDH (EC 1.4.1.2) is able to carry out the deamination of glutamate in higher plants. In the past it has been reported that overexpression of GDH genes results in reduction of plant bionmass lines (Tercé‐Laforgue et al., 2013).

The present study demonstrates that 100SDW in the desi chickpea MAGIC population is mainly determined by large number of small effect SNPs. Desi chickpea MAGIC population developed using eight founder lines and its genetic linkage map is a valuable genetic resource that could allow scientists and breeders to carry out genetic studies. Further, the genome‐wide associations identified for drought tolerance and the genes identified like *FRIGIDA*, TIFY 4B, E3 ubiquitin‐protein ligase RNF185‐like associated with key drought tolerance related traits can be used for improving these traits in chickpea breeding programs. In additions, markers can be designed for genes (CaTIFY4b) containing superior haplotypes for use in early generation selection for 100 seed weight or seed size in chickpea.

## AUTHOR CONTRIBUTIONS

**Mahendar Thudi**: Formal analysis; Investigation; Writing – original draft; Writing – review & editing. **Srinivasan Samineni**: Methodology; Resources. **Wenhao Li**: Formal analysis. **Martin P. Boer**: Formal analysis. **Manish Roorkiwal**: Writing‐review & editing. **Zuoquan Yang**: Formal analysis. **Funmi Ladejobi**: Data curation. **Chaozhi Zheng**: Formal analysis. **Annapurna Chitikineni**: Project administration. **Sourav Nayak**: Formal analysis. **Zhang He**: Formal analysis. **Vinod Valluri**: Formal analysis. **Prasad Bajaj**: Formal analysis. **Aamir W. Khan**: Data curation. **Pooran M. Gaur**: Methodology; Resources. **Fred van Eeuwijk**: Formal analysis; Writing‐review & editing. **Richard Mott**: Formal analysis. **Liu Xin**: Formal analysis; Resources; Supervision; Writing – review & editing. **Rajeev K. Varshney**: Conceptualization; Funding acquisition; Supervision; Writing‐review & editing.

## CONFLICT OF INTEREST STATEMENT

The authors declare that the research was conducted in the absence of any commercial or financial relationships that could be construed as a potential conflict of interest.

## Supporting information

Table S1 Summary of sequence statistics on founders and MAGIC lines.

Table S2 Summary of sequence data generated and alignment statistics on Founders of desi chickpea MAGIC population.

Table S3 Summary of sequence data generated and alignment statistics on desi chickpea MAGIC population.

Table S4 Summary of SNPs identified in case of individual MAGIC line.

Table S5 Proportion of heterozygous SNPs among founders.

Table S6 Pairwise SNPs identified between the founders.

Table S7 Distribution of founder specific unique SNPs in MAGIC population on different chromosomes.

Table S8 Proportion of founder specific unique SNPs in MAGIC population on different chromosomes.

Table S9 Details of the genetic map developed using the MAGIC population.

Table S10 Marker‐trait associations identified based on whole genome resequencing data and phenotyping of desi chickpea MAGIC lines.

Supporting Information

## Data Availability

The original contributions presented in the study are publicly available. This data can be found here: NCBI repository, BioProject ID PRJNA750634.
