# Supplementary material for: Whole genome resequencing and phenotyping of MAGIC population for high resolution mapping of drought tolerance in chickpea
Source: Plant Genome. 2023 Apr 30;17(1):e20333. doi: 10.1002/tpg2.20333 (PMC12806880; doi:10.1002/tpg2.20333)
Supplement: Supplementary file 1 [file TPG2-17-e20333-s011.pdf]

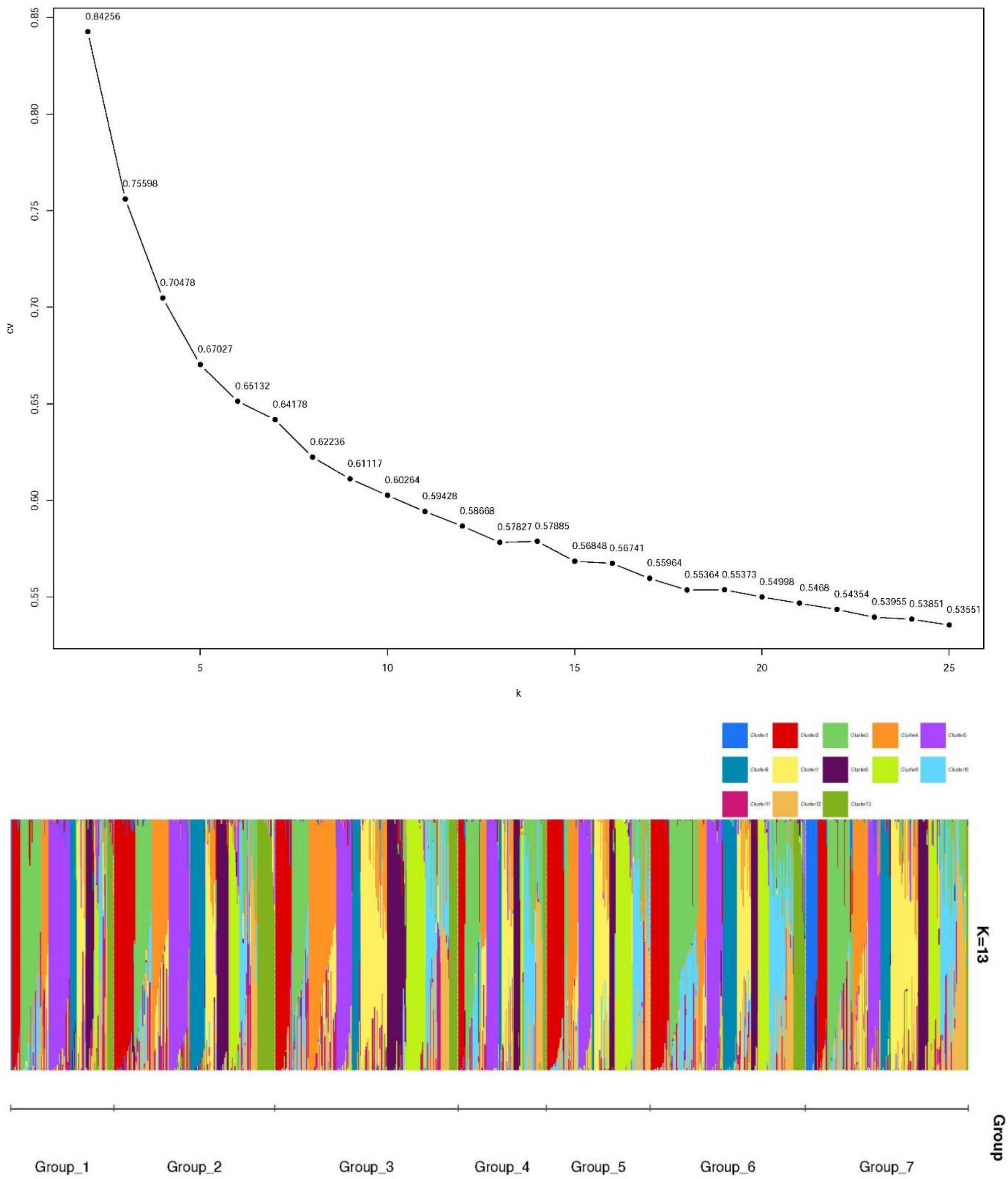

Supplementary Figure 1: Population structure. (A) Admixture analysis revealed 13 sub-populations in desi chickpea MAGIC population and (B) High admixture of alleles is evident. (C) PCA plot (left panel) and IBS-based kinship matrix (right panel) show no family structure

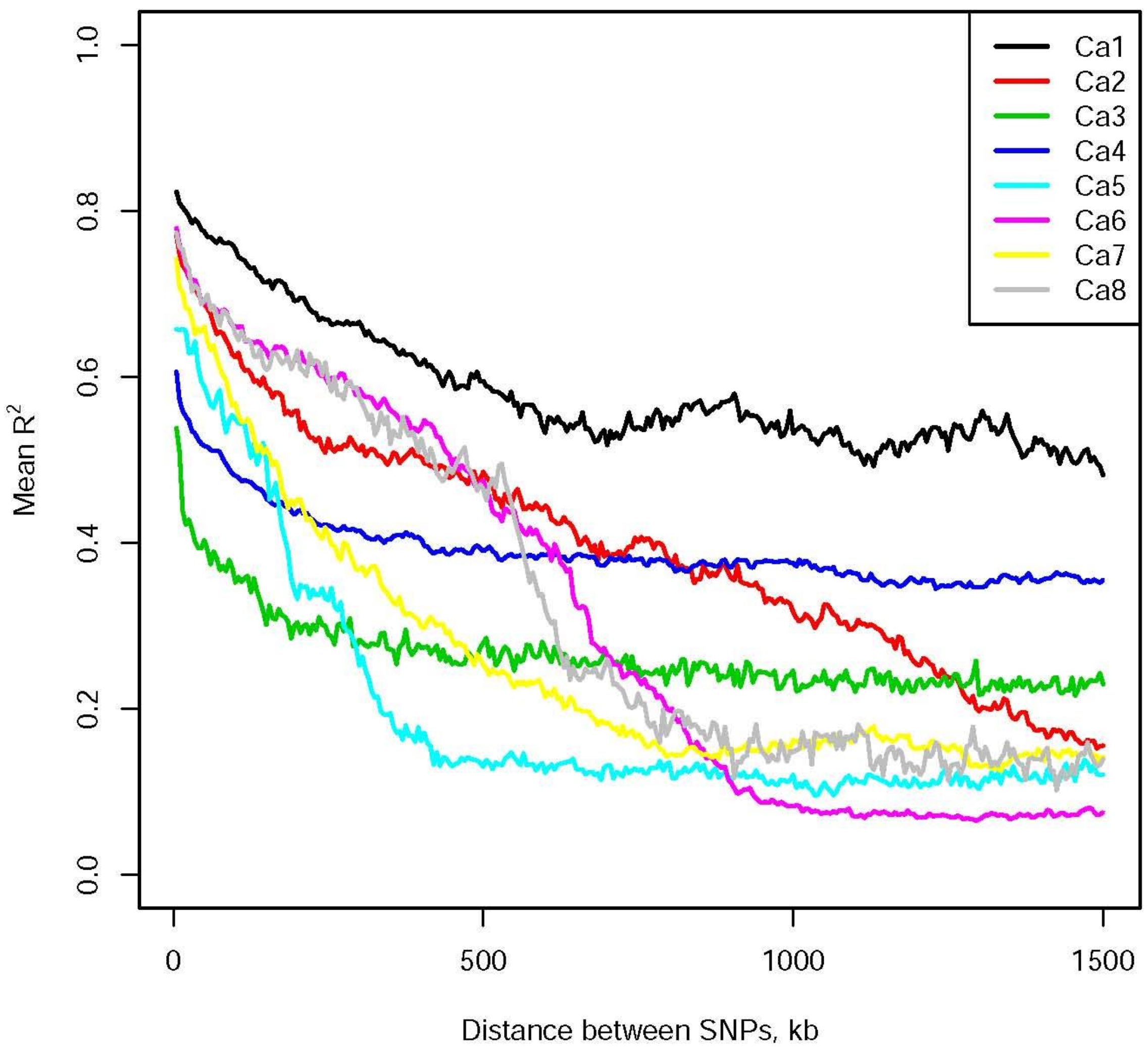

Supplementary Figure 2: Genome wide LD decay in MAGIC population. The LD decay varied between 0.5 Mb to 1.5 Mb among eight pseudomolecules.

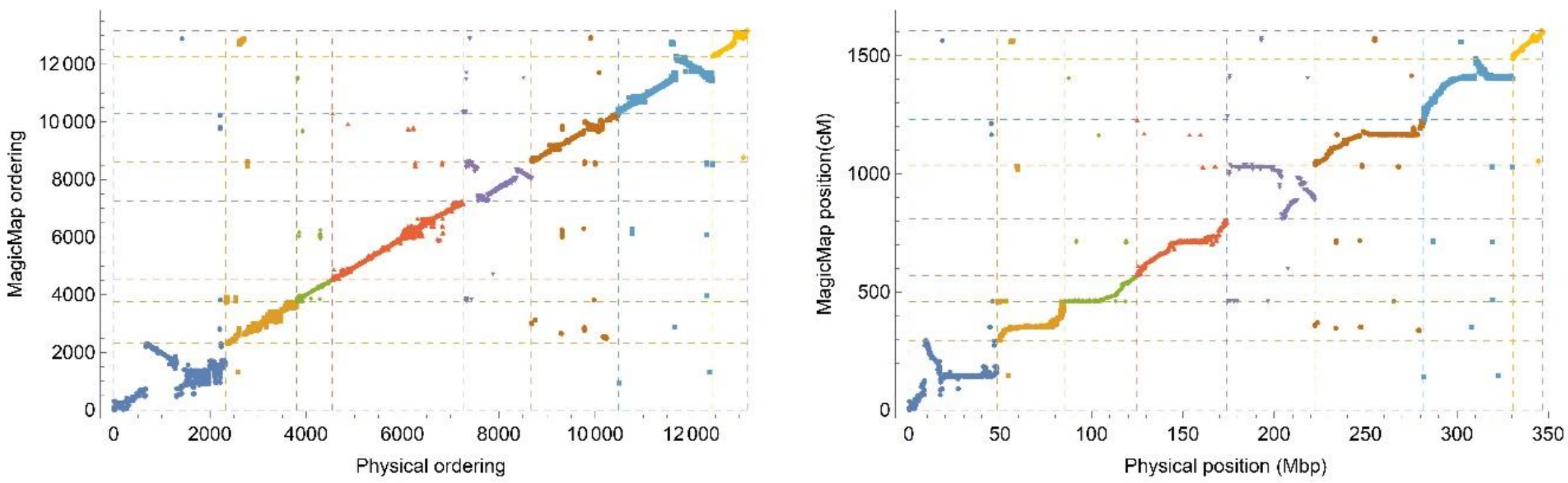

Supplementary Figure 3: Correspondence between physical and genetic map. Except for Ca1 and Ca8 high correspondence between physical map and genetic map was observed

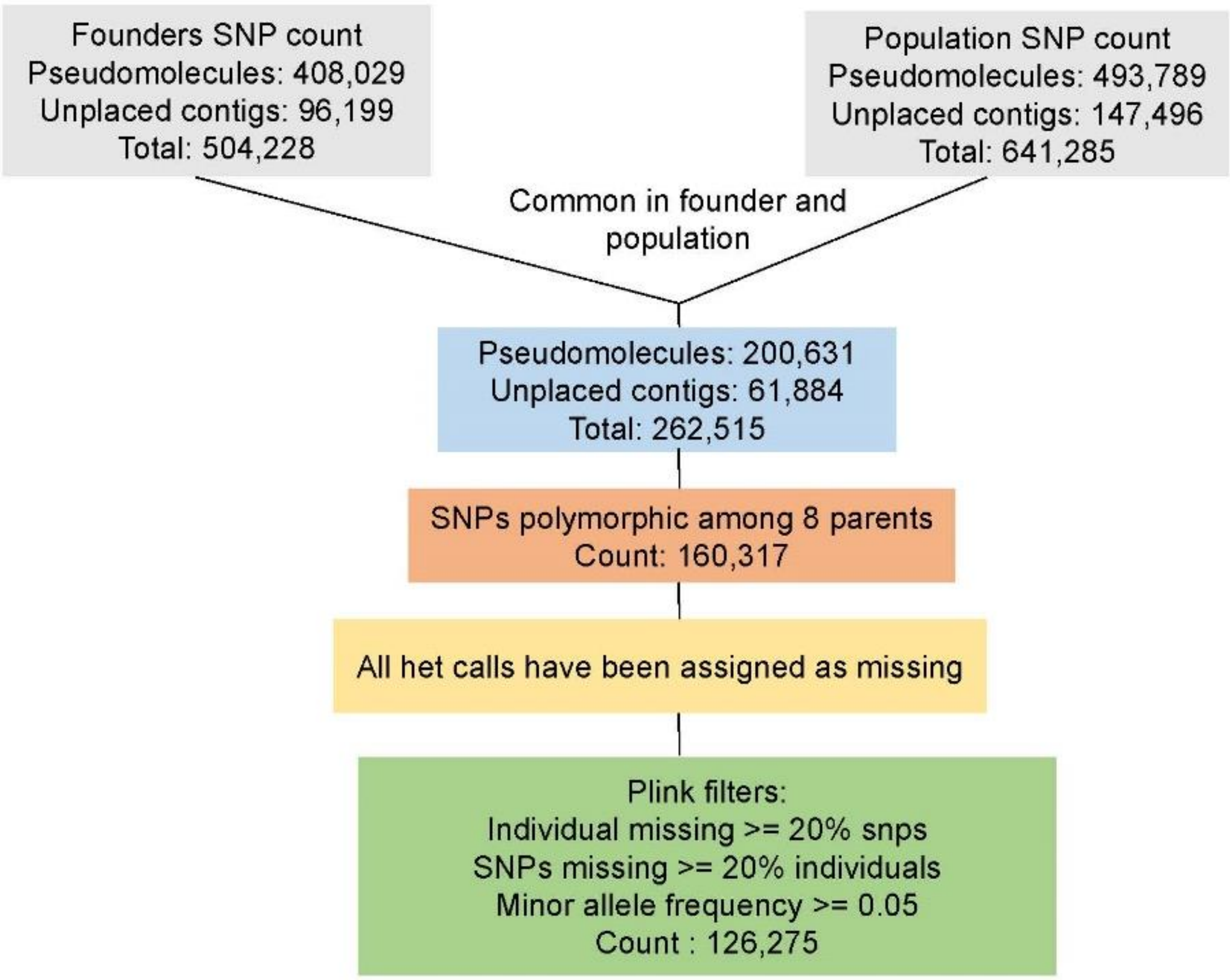

Supplementary Figure 4: Schematic representation of filtration of SNPs for genome wide association analysis
